# Team talk and team activity in simulated medical emergencies: a discourse analytical approach

**DOI:** 10.1186/s13049-016-0325-1

**Published:** 2016-11-14

**Authors:** Stine Gundrosen, Ellen Andenæs, Petter Aadahl, Gøril Thomassen

**Affiliations:** 1 Medical Simulation Centre, Trondheim, Norway; 2 Department of Anesthesia and Intensive Care Medicine, St. OIavs Hospital, Trondheim University Hospital, Trondheim, Norway; 3 Department of Circulation and Medical Imaging, Norwegian University of Science and Technology, Trondheim, Norway; 4 Department of Language and Literature, Norwegian University of Science and Technology, Trondheim, Norway

**Keywords:** Emergency medicine, Patient safety, Interdisciplinary teams, Communication, Patient simulation, Qualitative research

## Abstract

**Background:**

Communication errors can reduce patient safety, especially in emergency situations that require rapid responses by experts in a number of medical specialties. Talking to each other is crucial for utilizing the collective expertise of the team. Here we explored the functions of “team talk” (talking between team members) with an emphasis on the talk-work relationship in interdisciplinary emergency teams.

**Methods:**

Five interdisciplinary medical emergency teams were observed and videotaped during *in situ* simulations at an emergency department at a university hospital in Norway. Team talk and simultaneous actions were transcribed and analysed. We used qualitative discourse analysis to perform structural mapping of the team talk and to analyse the function of online commentaries (real-time observations and assessments of observations based on relevant cues in the clinical situation).

**Results:**

Structural mapping revealed recurring and diverse patterns. Team expansion stood out as a critical phase in the teamwork. Online commentaries that occurred during the critical phase served several functions and demonstrated the inextricable interconnections between team talk and actions.

**Discussion:**

Discourse analysis allowed us to capture the dynamics and complexity of team talk during a simulated emergency situation. Even though the team talk did not follow a predefined structure, the team members managed to manoeuvre safely within the complex situation. Our results support that online commentaries contributes to shared team situation awareness.

**Conclusions:**

Discourse analysis reveals naturally occurring communication strategies that trigger actions relevant for safe practice and thus provides supplemental insights into what comprises “good” team communication in medical emergencies.

## Background

Analyses of adverse events in medical emergency situations have emphasized the importance of good communication, and several reports conclude that communication errors can jeopardise patient safety [1–5]. Team communication is particularly important for coordinating responses to medical emergencies [6–8]. These situations are characterized by high complexity due to the rapidly changing state of the patient and the attendance of several experts with different medical specialties. Interdisciplinary medical emergency teams are composed of the individuals that are on call at the time rather than being a predetermined group of individuals [9, 10]. Although all team members have the same goal i.e. to offer the patient the best available treatment, each person assesses and approaches the situation based on their own individual professional expertise [10, 11]. Thus, to optimize treatment and to coordinate team activities, communication amongst team members, termed “team talk”, is crucial for utilizing the collective expertise during team interactions. Communication skills are highly emphasized both in emergency team training and in the assessment of team performance [11–16]. Recommendations for standardized communication, including closed loop communication, have been obtained mainly from work in the defence and aviation communities. However, the extent to which such communication strategies is implemented in medical practice remains unclear [17–19]. An additional concern is that the functions of medical emergency team talk—that is, the relationship between what is said and what is done—have remained more or less unexplored [10, 20, 21].

Qualitative discourse analysis is an inductive linguistic methodological approach to studying the interconnections between naturally occurring language and professional practices in an attempt to reveal the structural and interactional organization of the speech that takes place in certain situations. This approach pays particular attention to the micro level of interactions and to how decisions and actions can be considered interactional achievements based on negotiations by the team members [22, 23]. In the healthcare context, discourse analysis is used to investigate the structure and interactions between patients and clinicians in general medical practices, in genetic counselling and consultations in the emergency department [24–26]. It is also used to analyse shift handoffs and to identify communication patterns that are linked to collaboration during preoperative team briefings [27, 28]. Transcription is a decisive element in discourse analysis. Transcription offers a way to translate the content and structure of an interaction into a written format that helps the analyst notice details that are not readily apparent through observation, looking and listening. Transcription is thus an important tool for capturing interactional dynamics and for identifying patterns and variety across a corpus of data [29]. “Online commentaries” are utterances that frequently occur during a physical examination in patient-physician consultations. Online commentaries describe or evaluate what the physician is observing at that exact moment, and they both reassure the patient and contribute to the physician’s evaluation of the patient’s problems [30]. In the context of team communication, online commentaries are the way that team members share information of their real-time observations and assessments of observations based on relevant cues in the clinical situation [31–33]. Online commentaries are thus elements in team coordination and team adaption, which is associated with better team performance and which can impact patient safety [34–36].

Medical simulation has become an important arena for teaching and studying teamwork [37–39]. Simulation provides an opportunity to present the same patient scenario to multiple medical teams. *In situ* simulation allows teams to practice their response in a known environment with familiar medical equipment, making the simulation more realistic to the participants [39, 40]. *In situ* simulation provides a unique opportunity to explore the connections between what is said and what is done (what actions are taken).

To our knowledge, discourse analysis has not been used previously to study the functions of talk in interdisciplinary ad hoc emergency teams. To understand more about the interconnections between team talk and actions, we introduced the use of this analytical approach to 5 authentic teams during *in situ* simulation training in the emergency department at the hospital.

The aim of this study was to investigate functions of team talk with an emphasis on talk-work relationship by analysing the interconnections between online commentaries and actions in a communicatively and medically critical phase of the teamwork. Data was collected during *in situ* simulation training for interdisciplinary medical emergency teams.

## Methods

Data were collected from March to September 2012 during full-scale *in situ* simulations in the emergency department of a university hospital in Norway. The study was registered and approved by the Data Protection Official for Research at the hospital and by the managing authorities at the emergency department where the data collection took place. To capture the interconnections between team talk and actions in interdisciplinary emergency teams, we chose to simplify and standardize the emergency setting as much as possible through *in situ* simulation. The simulation was part of a joint internal curriculum that involved three hospital departments (the emergency, internal medicine and anaesthesia departments). The learning objective was to establish an acute medical response team and new routines for treating patients who were admitted to the hospital with critical illness. All *in situ* simulations were videotaped for post-scenario debriefing. One of the authors (EA) had a background in applied linguistics, so to familiarize this author with the simulation situation, this author observed 12 full training sessions and recorded team activities using field notes. The first 4 sessions were used only for familiarization purposes; however, the participants in the other 8 sessions were informed about the study, and we requested permission from them to transcribe and analyse the videotapes after the simulation training. All participants provided consent, and none chose to withdraw from the study.

**Fig. 1:**
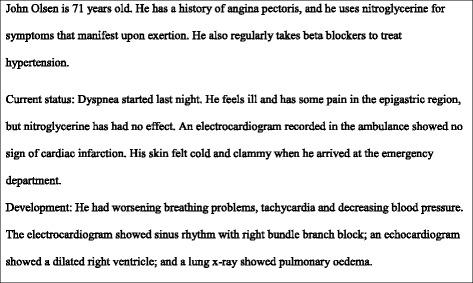
Case history (simulation scenario)

The *in situ* simulation was conducted with a man-sized patient simulator (SimMan 3G, Laerdal Medical, Stavanger, Norway). The scenario was pre-programmed (Fig. 1), and the program was run by a simulation facilitator. This facilitator also provided the patient’s verbal responses through a wireless sound transmission system. The central and peripheral pulses were palpable, and chest movements were observable. The participants were also able to observe the patient’s physical deterioration with standard monitoring equipment that is available in the emergency department. A second simulation facilitator began videotaping and then played the role of a paramedic who was handing the patient over to the team of participants. After the handover, this second facilitator provided each team with the patient’s test results and answered questions regarding the simulation throughout the scenario.

Of the 8 videotapes, 3 could not be transcribed in full due to technical reasons thus only 5 videos was availiable for analysis. The videos involved a total of 30 healthcare workers on 5 different acute medical response teams. The members of each team (Teams 1–5) were representative of the composition of a typical ad hoc team and were composed of individuals that work together at the hospital. The scenario (Fig. 1) began with a report that was handed over to the specialty registrar or physician consultant on call from the internal medical department (Phys1) and to two experienced emergency department nurses (EDnu1 and EDnu2). Next in the simulation, the patient’s situation deteriorated, and the physician called for assistance. This request activated the response team, which consisted of one anaesthesiologist (AN), one nurse anaesthetist (nuAN), and another specialty registrar or physician consultant from the internal medical department (Phys2). Notably, a medical student was present on Team 3, and Team 2 had to manage without the anaesthesiologist, who was occupied elsewhere during the time of the simulation. Thus, the scenario represented the realistic hospital admission of a critically ill patient. The training sessions ended shortly after the patient was intubated.

### Analysis

The authors had extensive experience from clinical emergency situations, medical simulation (SG and PA) and applied linguistics (EA and GT). We followed standard procedures to prepare for and to conduct an analysis of the recorded data. First, all 5 videos were viewed repeatedly. Second, the recorded (videotaped) data were transcribed in detail following established conventions that were developed for researching authentic interactions. The transcription was done in a format that was developed to portray the interactional architecture systematically by marking parallel talk (separate but concurrent verbal exchanges), pauses and non-verbal activities. Comments on the interactions between participants, interactions with the patient and interactions with the facilitator were extracted from the field notes and videos and added to the transcripts. Thus, the data included both team talk and the corresponding actions. All authors reviewed the transcripts for accuracy regarding the team talk, the interactional activities and the medical activities. To foster reflexivity and avoiding preconceptions affecting the results, the four authors performed analyses together and discussed the interpretations critically [41].

In recognition of the relationship between talk and work, we introduced activity type analysis which is a version of discourse analysis that focuses on the flexible relationship between talk and function in the communicative activity type [42]. The simulation sessions were considered as realizations of a goal-oriented communicative activity type. Consequently, every utterance could be analysed as part of a specific communicative phase that was recognizable by specific actions and interactions [43]. First, the talk was mapped into general recursive structural components—key activity phases—that were identified as an overarching structure that had associated sub-phases across all teams. Second, a sequential approach was used to deal with specific medical and interactional issues that was addressed by the team (such as when to expand the team, how to respond to observations, how to distribute tasks and responsibilities and the timing of the interventions). These issues formed recognizable communicative phases. These phases comprised the sequential organisation of team talk, which is linked to various identifiable functions [44]. Third, we used activity type analysis to identyfy a medically and communicatively critical phase of the teamwork. Recognizing the importance of speaking up about relevant individual real-time observations and assessments in interdisciplinary teamwork, we analysed the function of utterances that can be characterized as online commentaries.

## Results

Here we present excerpts that were selected to illustrate the data and that support our findings. The utterances are numbered according to their sequence in the team talk. All transcripts were anonymized and were translated from Norwegian after the analysis.

### Key activity phases

Multiple careful viewings of the videos revealed three overall key activity phases and associated sub-phases that were present in all of the simulations. Each of the three phases was tied to a clinical process and reflected the state of the simulated patient. Phase 1, the *opening phase*, consisted of a greeting, a summary of the case history, an assessment related to the case history and a call for extra help. Phase 2, the *core activity*, included an expansion of the team, a new case/patient history report, an assessment of the information, discussions and treatment. Phase 3, the *closing phase*, included patient monitoring, ordinations and discussions of follow-ups.

Excerpt A demonstrates the talk-work relationship in one of the teams right after tracheal intubation (core activity).

**Excerpt A**

**Table:** 

Utterance	Speaker	Talk	Action
472	Phys2	Mhm. Could it be a ^1^[CNS (central nervous system) problem?]^1^	Phys2 looks at Phys1.
473	Phys1	^1^[And of course]^1^ I’ll need blood samples. What?	Phys1 looks and points to EDnu2 standing by the documentation desk. EDnu1 looks at Phys1. Phys1 then looks at Phys2.
474	Phys2	Could it be a CNS problem?	Phys2 looks at Phys1.
475	Phys1	Well, he was awake ^2^[when he arrived and he was in pain]^2^. No, yes.	nuAN is fixating the tube. AN is performing bag-tube ventilation.
476	Phys2	^2^[There wasn’t any paresis, was there?]^2^	Phys2 looks at Phys1.
477	nuAN	Should we insert a ^3^[nasogastric tube?]^3^	nuAn is standing beside AN.
478	Phys1	^3^[Yes, but didn’t get a chance to perform a]^3^ ^4^[full neuro exam.]^4^	EDnu1 draws blood.
479	AN	^4^[We may insert a nasogastric tube.]^4^	AN looks down on the patient’s head and nods
480	Phys2	No, OK.	nuAN walks out of view to the right.
481	Phys1	Eh ^5^[X X X.]^5^	
482	AN	^5^[The pupils are still X.]^5^	AN leans over the patient’s head.
483	Phys2	Could we place a urine catheter if possible?	Phys2 looks down on the patient’s head.
484	AN	This pupil reacts, but the other one is hardly reacting.	AN, Phys1 and Phys2 lean over the patient’s head.
485	Phys1	No OK.	
486	AN	But are they equal ^6^[or not?]^6^	Phys1 looks out of the field of view to the left.
487	Sim	^6^[Yes they]^6^ are supposed to be equal.	EDnu1 looks at EDnu2.
488	nuAN	^6^[nasogastric tube?]^6^	nuAN walks toward the rear of the bed and speaks to EDnu2.

**Transcript key**: X = not audible; ^2^[words]^2^ = overlapping speech (the numbers indicate the order of the nearby overlap)

Excerpt A is an example of how overlapping dialogs and parallel talk mirror the complexity of the interactions and the parallel ongoing activities in the interdisciplinary emergency team. In this short sequence of team talk and corresponding actions, the team members, and even the simulation facilitator, were involved in multiple interactions and practical tasks. Sometimes the team members switched their focus from one utterance to the next, and sometimes they switched their focus even within the same utterance (e.g. Excerpt A, u 473). Frequently two or more team members talked simultaneously, and sometimes one team member was communicative and interactionally involved in more than one issue at the same time. One example of this is when AN was deciding whether to place a nasogastric tube and assessing the patient’s neurological status while also ventilating the patient.

Some utterances were simulation-related in that they contained information that normally is observable in real-life situations but that was not clear in the simulated scenario due to technical issues connected to the simulation or to the mannequin (i.e., Excerpt A u 486 and u 487).

### Online commentaries

The structural analysis drew our attention to the team expantion in phase 2 (the core activity). As medical experts joined the team, they became engaged in working on the patient, and the situation demanded concurrent attention to several tasks simultaneously. The patient’s history and status were repeated as each new team member arrived. In addition, the deteriorating status of the patient had to be managed, and the team had to be (re)organised by distributing tasks and responsibilities. This phase stood out both in clinical and communicative ways that were identifiable in videos and transcriptions; thus, the interval between calling for assistance until the time at which all team members were involved in the activity was considered a medically and communicatively critical phase. Through activity analysis at the micro level, we identified several different functions of online commentaries during this phase of the teamwork. Online commentaries are indicated in bold in the excerpts below.

**Excerpt B** (while Phys1 is reporting the patient’s history and status to new team members)

**Table:** 

Utterance	Speaker	Talk	Action
104	Phys1	^2^[Chest]^2^. ^3^[Short of breath and X X]^3^ ^4^[X X. It]^4^ ^5^[It’s gurgling X.]^5^	Phys1 looks at the patient, puts down the ECG sheet and walks toward the patient's head.
105	EDnu1	^3^[The patient’s name is John.]^3^	EDnurse1 looks at nuAN.
106	nuAN	^4^[How old is John?]^4^	nuAN looks at EDnu1.
107	EDnu1	^5^[John is]^5^	EDnu1 walks to the desk, where the patient’s record is located.
108	AN	^5^[I turned up the oxygen.]^5^	AN comes to the bed from the left where the oxygen flowmeter is positioned, looking down and reaching for her stethoscope. Phys1 looks at AN.
109	Phys2	^5^[OK.]^5^	
110	Phys1	And he is hypotensive	Phys1 looks at Phys2.
111	AN	^6^[But you]^6^ hear coarse rattling sounds.	AN looks at Phys1.
112	Phys1	I think I hear coarse rattling sounds ^6^[X X X.]^6^	Phys1 touches the patient’s ribcage.
113	X	mm	
114	Phys2	^7^[He’s getting fluid]^7^	Phys2 is looking at Phys1.
115	nuAN	^7^[He has]^7^ falling saturation.	nuAN looks at the monitor at the right. AN turns and looks at the monitor at the right.
116	Phys1	Yes.	
117	AN	^7^[Yes]^7^ ^8^[I turned up]^8^ the oxygen a bit.	
118	EDnu1	^8^[Yes.]^8^	
119	X	^8^[He had that yes.]^8^	
120	EDnu1	He has two IV cannulas. His pressure was a bit low. Blood pressure was 90 over 40.	EDnu1 points to the patient’s hand before he points up toward the monitor. EDnu1 then goes to the desk where the patient’s journal is located.
121	X	Mm	
122	Phys2	There ^9^[is fluid going]^9^ right?	Phys2 looks at Phys1 and points to the patient.
123	AN	^9^[But eh.]^9^	
124	EDnu1	Eh ^10^[two Ringers are going.]^10^	EDnu1 stands by the desk, where the patient’s journal is located and looks in the direction of the patient.
125	Phys1	^10^[Yes it is going in fact X X]^10^ I think.	Phys1 looks at and touches the IV fluid hanging over the patient’s chest.

**Transcript key**: X = word not audible; ^2^[words]^2^ = overlapping speech (the numbers indicate the order of the nearby overlap)

The report by Phys1 on the patient’s status in Excerpt B led to AN managing the oxygen. Upon hearing Phys1’s comment regarding the patient’s hypotension, AN responded to Phys1 by asking about auscultation. Thus, the online commentary “and he is hypotensive” led to a joint construction of tasks and to the distribution of responsibilities between AN and Phys1. The nuAN’s online commentary regarding her assessment of the patient’s blood oxygen saturation level triggered a report on an action from AN that acknowledged nuAN’s observation (u 117). In Excerpt B, Phys2’s commentary about ongoing fluids led EDnu1 to assess the available IV access points; this was framed as an online commentary and was followed by a statement on the patient’s blood pressure (u 120).

**Excerpt C** (AN not present)

**Table:** 

Utterance	Speaker	Talk	Action
197	nuAN	Eh, his saturation is falling. I think I have to assist him with his ventilation.	nuAN is looking at the monitor, holding a CPAP-mask to the patient’s nose and mouth and then looks around the room.

Excerpt C illustrates how nuAN’s online commentary comes across as an argument for a decision.

**Excerpt D** (while Phys1 is reporting the patient’s history and status to new team members)

**Table:** 

Utterance	Speaker	Talk	Action
91	AN	^1^[But his saturation is low.]^1^	AN looks at the monitor, and AN and nuAN both move toward the patient’s head.
92	Phys1	Yes he has received 15 l of O_2_. We should switch to a mask with a ^2^[reservoir.]^2^	Phys1 looks in direction of nuAN and AN.
93	AN	^2^[X X]^2^. (1.5) Take this one here instead. Let’s see can you connect it?	AN takes a bag-mask ventilator from the wall. AN looks at nuAN.

**Transcript key**: X = word not audible; (number) = pause, with the number indicating seconds; ^2^[words]^2^ = overlapping speech (the numbers indicate the order of the nearby overlap)

Phys1is reporting the patient’s history to new team members when AN comments on the patient’s low oxygen saturation level. AN’s online commentary was treated by Phys1 as an indirect instruction to change the oxygen treatment.

**Excerpt E**

**Table:** 

Utterance	Speaker	Talk	Action
123	nuAN	There is no saturation.	nuAN looks at the monitor on the right.
124	AN	Let’s see.	
125	EDnu1	No it fell [off.]	EDnu1 picks up the saturation probe from the floor.

**Transcript key**: [words] = overlapping speech

nuAN’s online commentary in Excerpt E regarding the missing oxygen saturation value elicited a response from EDnu1, who then re-established the monitoring probe used to measure oxygen saturation. This is an example of how an online commentary can be used to get things done without asking directly.

**Excerpt F**

**Table:** 

Utterance	Speaker	Talk	Action
142	AN	^1^[Does he have a pulse?]^1^	AN looks at Phys1. Phys1 moves to palpate for a carotid pulse. Phys2 palpates the patient’s groin.
143	Phys1	Yes.	
144	AN	Does he have a pulse? (3.0) Yes, his pulse is 77 X ^2^[yes X 77.]^2^	AN looks at the monitor at the right.
145	Phys1	^2^[Yes, he has a pulse.]^2^	Phys2 palpates for a carotid pulse and looks at the patient’s chest.
146	AN	Yes, 77. His saturation is going up. But I still think we have to ^3^[intubate him.]^3^	AN looks at the monitor on the right.
147	nuAN	^3^[Intubate him yes.]^3^	nuAN looks at AN.
148	Phys1	^3^[Intubate him yes.]^3^	Phys1 looks at AN.

**Transcript key**: X = word not audible; (number) = pause, the number indicates seconds; ^2^[words]^2^ = overlapping speech (the numbers indicate the order of the nearby overlap);

AN had previously proposed intubating the patient. In Excerpt F, AN implicitly asked the team members to assess the patient’s blood pressure and pulse before going ahead with the intubation. AN and Phys1 and Phys2 took action. AN and Phys1 provided online commentaries as responses to the request for information by AN.

**Excerpt G** (while Phys1 is reporting the patient’s history and status to new team members)

**Table:** 

Utterance	Speaker	Talk	Action
213	EDnu2	^1^[His saturation is falling.]^1^	EDnu2 looks at the monitor on the right.
214	Phys1	Yes that, yes. And suspicion of pulmonary oedema. He has crackling sounds in the lungs. Eh. And is slightly clammy peripherally. The blood gas result says metabolic ^2^[acidosis.]^2^	Phys1 looks quickly at the monitor and then at AN.

**Transcript key**: ^2^[words]^2^ = overlapping speech (the numbers indicate the order of the nearby overlap)

The online commentary from EDnu2 regarding the patient’s falling oxygen saturation level triggered a complementary report on the patient’s status.

## Discussion

In this study, we transcribed verbatim the speech of the members of five interdisciplinary teams during an *in situ* simulation of the hospital admission of a critically ill patient. We then analysed the team talk. Three key activity phases, i.e. the opening phase, the core activity phase and the closing phase, reflected the simulated patients’ clinical situations. When we used qualitative discourse analysis to evaluate the complex communications between team members, which at first seemed unstructured, patterns emerged in terms of the embedded structure and the targeted dialogue loops that recurred. These patterns showed the variations in team activities during the scenario. We found that team members could rapidly switch focus, sometimes even during a single utterance. This, together with frequently overlapping dialogues and parallel talk, illustrated the ongoing activity, the dynamics and the complexity of the teamwork. Through activity analysis, we found that the online commentaries served a number of different functions during the critical phase of team expantion.

The transcription of team talk and activity in these five teams provided the opportunity to study team interactions in slow motion. In this study we found evidence of the inextricable interdependency between communication in interprofessional emergency teams and team activity, supporting Roberts (2005) who claims: “In institutional encounters, talk is work” [22].

Structural mapping of team talk revealed recurring patterns on the one hand and diversity among the teams on the other hand. We captured what appeared to be the prototypical character of all teams and also the variety within and among the teams. The team talk did not follow a predefined structure such as that used for standardized cockpit communication; nevertheless, all of the teams appeared to have a common understanding of what to talk about. This mutual understanding can be viewed as a pattern that arose from common goals and methods [44]. The flow of the team talk shows the teamwork dynamics and how the team members had the flexibility and adaptability they needed to accomplish the expected tasks, which is important for high-level team performance [33, 34, 45]. Although the team talk appeared to be unstructured, which could have some risks, the team members managed to manoeuvre safely within this complex situation by communicating with each other. In interprofessional emergency teamwork, team members have special responsibilities regarding their medical expertise. In addition, team interactions depend on team members’ individual non-technical skills, such as cognitive and social skills, which are vital for preventing medical errors in teamwork [11, 12]. In ad-hoc teams, cooperation begins the moment that members interact. Teamwork thus involves negotiations both in terms of the clinical process and in terms of cooperation within the team. In this study, we excluded clinical differences between patients by using an *in situ* simulation and standardized scenarios. Differences between the teams could thus be interpreted as diversity in team cooperation.

In medical emergencies, patient safety depends on decisions that are based on team members’ awareness and on their ability to take the right actions at the right times. Adaptive team performance depends on each team member’s ability to identify and assess relevant cues from the environment, share information and adjust the activity as the situation changes [33–35]. The meanings of words within a community of practice are negotiated, confirmed and completed for current purposes [46]. Even so, misunderstandings do occur in emergencies, and sometimes they result in life-threatening situations. To help prevent errors during a response to medical emergencies, communication patterns such as closed loop communication have been adapted from the defence and aviation sectors. Despite the extensive focus on the relationship between closed loop communication and patient safety in medicine, Härgestam (2013) found that closed loop communication was rarely used during trauma team training, even in high performing teams [17]. In addition, closed loop communication initiated by a team member rather than by the team leader could lead to communication overload [47]. Online commentaries are closely related to utterances that are termed “information-related talking to the room”, which refers to interpreting and sharing information that is associated with the further use of information-related talking to the room in high-performing anaesthesia teams [48]. Kolbe (2010) suggests that such utterances can be interpreted as contributing to shared team situation awareness, which facilitates adequate patient safety [48]. Our results support this interpretation. Although closed loop communication is an important communicative tool, we must expand our knowledge about what constitutes “good” communication in interdisciplinary emergency teams by using analytical tools, such as discourse analysis, that can reveal communication strategies within the relevant activity type that trigger actions that are relevant to safe practices.

Patient simulation is increasingly used in team communication training. By identifying the functions of online commentaries, we observed that the cues provided by the patient simulator were restricted; i.e., team members could see, feel and hear a limited number of cues. For instance, in our material, we found no online commentaries on patient behaviour (e.g. shivering), appearance (e.g. oedema) or skin signs (e.g. temperature, colour). Such cues were only available through the simulation facilitator and thus were not framed as online commentaries within the team. When conducting team simulation training and assessing teamwork during simulations, it is important to consider whether simulation itself might affect the talk-work relationship that is vital for team coordination and adaptability.

### Study limitations and strengths

Studying multiple authentic teams that are working with similar “patients” provided an opportunity to demonstrate the usefulness of discourse analysis for analysing communication in interdisciplinary ad hoc emergency teams. One limitation of this study was that the team talk was analysed for just five medical teams. Nevertheless, the amount of data was sufficient to investigate the functions of team talk with an emphasis on the talk-work relationship in simulated scenarios. Although we used sophisticated computerized mannequins, patient simulators have limitations because they have limited observable clinical changes. In addition, team talk might be affected by the knowledge that the situation was a simulation. Similarly, the knowledge that the members were being observed and studied may have affected participant performance during the simulation. Since it is unknown exactly how and to what extent this factor might affect performance [23], to avoid bias permission to transcribe and analyse the video recordings was requested after the simulation training was complete. To blend into the setting as much as possible, the observer was introduced as a communication specialist who was interested in health communications. The participants were informed that the observer had been instructed not to commingle in the simulation training. To avoid distraction, we used just one camera and one stand-alone microphone instead of lapel microphones. The resulting variable sound quality made transcription of some of the speech challenging; however, the field notes compensated sufficiently for these difficulties. Although it is widely recognized that body language is important in communication, analysing body language was not a specific aim of the present study [49, 50]. Nevertheless, connecting team talk to teamwork represents a new methodological approach that is important for researching communication in medical emergencies.

## Conclusion

In this study, we used qualitative discourse analysis to evaluate video-recorded, interdisciplinary emergency ad-hoc teams during a simulated emergency. Through discourse analysis, we were able to capture the dynamics and the complexity of team talk. This analysis revealed the key functions of the team talk and the inextricable interdependency between team talk and teamwork. Through structural mapping, we identified the essential dimensions of team talk that were related to the activity type. On one hand, recurrent patterns indicated prototypical characteristics; on the other hand, there were variations that reflected specific negotiations and diverse situations. Even though the team talk did not follow a predefined structure, the team members managed to manoeuvre safely within the complex situation. The analysis revealed the functions of online commentaries that were essential for team situational awareness and for coordination of teamwork as well as issues that might affect the talk-work relationship in simulation training. In general, discourse analysis reveals naturally occurring communication strategies that trigger actions that are relevant to safe practices; here, this analysis provided important supplemental information that is useful for pursuing good team communication in medical emergencies.

## Abbreviations

AN: AnaesthesiologistEDnu1: Experienced nurse from the emergency department
EDnu2: Experienced nurse from the emergency departmentnuAN: Nurse anaesthetist
Phys1: First specialty registrar or consultant from the internal medical department at the scene
Phys2: Second specialty registrar or consultant from the internal medical department at the scene
Sim: Simulation facilitator
